# Supplementary material for: A comparison of biomonitoring methodologies for surf zone fish communities
Source: PLoS One. 2023 Jun 14;18(6):e0260903. doi: 10.1371/journal.pone.0260903 (PMC10266690; doi:10.1371/journal.pone.0260903)
Supplement: S1 File — (DOCX) [file pone.0260903.s001.docx]

**Supplemental Methods**

Seawater for eDNA was collected after seine and BRUV surveys were completed, to minimize time for degradation during transport. We collected these samples, haphazardly spaced 40 to 50 meters apart, within the area surveyed by seine and BRUV in ~1.5 m of water. To minimize human DNA contamination, we conducted sample collection in a neoprene wetsuit using nitrile gloves, and thoroughly rinsed wetsuits with freshwater between collection sites to prevent carryover of eDNA among sites. We sealed samples in Ziplock bags and kept them on ice until reaching the laboratory. In the laboratory, we gravity filtered each seawater sample through a 0.2 µm Sterivex filter within four hours of sample collection to limit DNA degradation [1]. Filter times varied, but we filtered 0.5 L of seawater for each sample except for one sample replicate from Little Harbor (Catalina Island, Sample A) where it took > 2 h to filter 0.2 L of seawater (Table S1). Filters were stored at -20 °C for up to eight weeks prior to extraction at the University of California Los Angeles, Los Angeles, California, USA (UCLA).

We performed DNA extractions using a modified Qiagen DNAeasy Blood and Tissue kit (Qiagen Inc., Gernmantown, MD, USA). We followed manufacturer protocols as modified by Spens et al. [2], adding 80 µL proteinase K and 720 µL ATL buffer directly to the filter cartridges before overnight incubation in a rotating incubator at 56 ̊C. Final elution volumes were 100 µL.

We amplified teleost (bony fish) eDNA using the 12S MiFish Universal Teleost primers with linker modifications for Nextera indices (MiFish-U [3]). We used 25 μL reactions with 12.5 μL Qiagen Multiplex Taq PCR 2x Master Mix, 6.5 μL dH_2_0, 2.5 μL of each primer (2 μmol/L), and 1 μL template DNA [4]. We amplified elasmobranch (cartilaginous fish) eDNA using the MiFish-E primers [3] with linker modifications for Nextera indices. We used 25 μL reactions with 12.5 μL Qiagen Multiplex Taq PCR 2x Master Mix, 1.5 μL dH20, 5 μL of each primer (2 μmol/L), and 1 μL template DNA (total extracts eluted in 100 µL) [4]. For both primer sets, thermocycling employed a touchdown PCR profile consisting of an initial denaturation at 95 ̊C for 15 minutes, 13 denaturation cycles at 94 ̊C for 30 seconds each, beginning annealing at 69.5 ̊C for 30 seconds with every subsequent cycle temperature decreasing by 1.5 ̊C until reaching 50 ̊C, extension at 72 ̊C for 1 minute with 35 additional cycles at annealing temperature 50 ̊C and final extension at 72 ̊C for 10 minutes [4]. Each PCR included both positive and negative PCR controls; negative controls substituted molecular grade water in place of the DNA extraction, and we used either American alligator (*Alligator mississippiensis*) or dromedary (*Camelus dromedarius*), species non-native to California, for positive controls. To account for variation resulting from PCR bias [5,6], we performed all PCRs in triplicate, with each triplicate herein referred to as a technical replicate. All PCR results were visualized through electrophoresis using SybrGreen on a 2% agarose gel.

To create sequencing libraries, we attached index barcodes using the Nextera Unique Dual Index A, B, C and D Kits (Illumina, San Diego, CA, USA). Indexing reactions consisted of 25 μL reaction mixture with 12.5 μL Kapa HiFi Hotstart Ready Mix (Kapa Biosystems, Wilmington, MA, USA), 1.25 μL Nextera UD Index, and 10 ng of PCR product. Thermocycling parameters were: initial denaturation at 95 ̊C for 5 minutes, 5 cycles of denaturation at 98 ̊C for 20 seconds, annealing at 56 ̊C for 30 seconds, extension at 72 ̊C for 3 minutes, and final extension at 72 ̊C for 5 minutes [4]. We confirmed correct size of indexed PCR products through electrophoresis on a 2% agarose gel, cleaned them with Serapure magnetic beads [7], and quantified DNA concentrations using the high sensitivity Quant-iT dsDNA Assay Kit (Thermofisher Scientific, Waltham, MA, USA) on a Victor3 plate reader (Perkin Elmer, Waltham, MA, USA). We then pooled all samples in equimolar concentrations, resulting in two libraries, one for MiFish-U and one for MiFish-E. We sequenced these libraries on a NextSeq PE 2 x 150 bp mid-output at the Technology Center for Genomics & Bioinformatics (UCLA) with 20% PhiX added to all sequencing runs.

*Bioinformatics*

We processed the resulting eDNA metabarcoding sequences using the *Anacapa Toolkit* (version 1) [4], conducting quality control, amplicon sequence variant (ASV) parsing, and taxonomic assignment. We processed each sequencing library twice using two different barcoding reference libraries. First, to assign taxonomy to marine mammalian and avian species, we used the *CRUX*-generated-*12S* database, comprised of reference barcodes for all publicly available *12S* barcodes [8]. Second, we used a curated metabarcoding database specific to California coastal marine fish to generate taxonomic assignments for fishes [8]. We employed a Q score cutoff of 30 and Bayesian taxonomic cutoff score of 60 following the methods of Gold et al. [8]. The resulting taxonomic tables were transferred into *R* for further processing [9].

Given that small levels of background contamination are common in metabarcoding data, we employed a multifaceted decontamination approach developed by Kelly et al. [10] to remove field contamination, lab contamination, and index hopping [11,12]. Through this process we implemented a hierarchical site occupancy modeling framework to distinguish occupancy rates across multiple sample and technical replicate detections [10,13]. Only ASVs detected in at least two technical replicates in a site were kept. From these processes, we obtained decontaminated eDNA species-by-sample community tables with counts of total sequence reads.

Next, to examine relative abundance, we transformed the eDNA read counts into eDNA index scores according to Kelly et al. [10]. To compute the eDNA index, which normalizes the read count per technical PCR replicate per species, we calculated the relative abundance of each species in each technical PCR replicate by dividing the total number of reads of each species by the total number of reads in each technical PCR replicate. The relative abundance was then divided by the maximum relative abundance for a given species across all samples, yielding the eDNA index score, which ranges from 0 to 1 and allows for comparisons of relative abundance for specific taxa across samples. We note that the eDNA index was calculated per species, meaning reads from multiple ASVs assigned to the same species were summed, including multiple ASVs from the two MiFish markers employed.

*Occupancy Model*

Each pattern of occurrence for a given taxon within a given site was considered a case (e.g. 2 detections out of 4 seine tows). Each unique model was run 10 times in order to filter out cases in which the model converged into a local maximum. For eDNA approaches, we employed a hierarchical model accounting for multiple technical replicates nested within bottle replicates for the eDNA surveys. In addition, for eDNA methods, we summarized the number of occurrences of each case and ran each case through a separate occupancy model to reduce computational time.

We used the same reasonably informative priors for parameter estimations for each survey method. First, we assume that our methods do a reasonably good job of detecting species, if the species are present [13,14]. Thus, true positive probability (P_11_) was modeled with priors from a left-skewed beta distribution where alpha = 2 and beta = 2. Occupancy probability (P_si_) modeled with using weak priors from a left-skewed beta distribution where alpha = 2 and beta = 2 assuming that most species are common across sites in this study. Lastly, we assumed that the false-positive rate of detection is unlikely to approach the true-positive rate. Thus, false positive probability (P_10_) was modeled with priors from a right-skewed beta distribution where alpha = 1 and beta = 10. Stan occupancy models are included in Supplemental Materials.

References

1. Minamoto T, Miya M, Sado T, Seino S, Doi H, Kondoh M, et al. An illustrated manual for environmental DNA research: Water sampling guidelines and experimental protocols. Environ DNA. 2020.

2. Spens J, Evans AR, Halfmaerten D, Knudsen SW, Sengupta ME, Mak SST, et al. Comparison of capture and storage methods for aqueous macrobial eDNA using an optimized extraction protocol: advantage of enclosed filter. Yu D, editor. Methods Ecol Evol. 2017;8: 635–645. doi:10.1111/2041-210X.12683

3. Miya M, Sato Y, Fukunaga T, Sado T, Poulsen JY, Sato K, et al. MiFish, a set of universal PCR primers for metabarcoding environmental DNA from fishes: detection of more than 230 subtropical marine species. R Soc Open Sci. 2015;2: 150088. doi:10.1098/rsos.150088

4. Curd EE, Gold Z, Kandlikar GS, Gomer J, Ogden M, O’Connell T, et al. Anacapa: an environmental DNA toolkit for processing multilocus metabarcode datasets. Methods Ecol Evol. 2019;10: 1469– 1475. doi:https://doi.org/10.1111/2041-210X.13214

5. Kelly RP, Shelton AO, Gallego R. Understanding PCR processes to Draw Meaningful conclusions from environmental DNA Studies. Sci Rep. 2019;9: 1–14. doi:https://doi.org/10.1038/s41598-019-48546-x

6. Doi H, Fukaya K, Oka S, Sato K, Kondoh M, Miya M. Evaluation of detection probabilities at the water-filtering and initial PCR steps in environmental DNA metabarcoding using a multispecies site occupancy model. Sci Rep. 2019;9: 3581. doi:https://doi.org/10.1038/s41598-019-40233-1

7. Faircloth BC, Glenn TC. Protocol: Preparation of an AMPure XP substitute (AKA Serapure). DOI. 2014;10: J9MW2F26.

8. Gold Z, Curd E, Goodwin K, Choi E, Frable B, Thompson A, et al. Improving Metabarcoding Taxonomic Assignment: A Case Study of Fishes in a Large Marine Ecosystem. 2021.

9. R Core Team. R: A Language and Environment for Statistical Computing. Vienna; Austria; 2020.

10. Kelly RP, Gallego R, Jacobs-Palmer E. The effect of tides on nearshore environmental DNA. PeerJ. 2018;6: e4521. doi:https://doi.org/10.7717/peerj.4521

11. Costello M, Fleharty M, Abreu J, Farjoun Y, Ferriera S, Holmes L, et al. Characterization and remediation of sample index swaps by non-redundant dual indexing on massively parallel sequencing platforms. BMC Genomics. 2018;19: 332.

12. Goldberg CS, Turner CR, Deiner K, Klymus KE, Thomsen PF, Murphy MA, et al. Critical considerations for the application of environmental DNA methods to detect aquatic species. 2016 [cited 20 Mar 2019]. doi:10.1111/2041-210X.12595

13. Chambert T, Pilliod DS, Goldberg CS, Doi H, Takahara T. An analytical framework for estimating aquatic species density from environmental DNA. Ecol Evol. 2018;8: 3468–3477. doi:10.1002/ece3.3764

14. Gold Z, Wall AR, Curd EE, Kelly RP, Pentcheff ND, Ripma L, et al. eDNA metabarcoding bioassessment of endangered fairy shrimp (Branchinecta spp.). Conserv Genet Resour. 2020;12: 685–690.

15. Longo GC, Bernardi G, Lea RN. Taxonomic revisions within Embiotocidae (Teleostei, Perciformes) based on molecular phylogenetics. Zootaxa. 2018;4482: 591–596.

16. Duke EM, Burton RS. Efficacy of metabarcoding for identification of fish eggs evaluated with mock communities. Ecol Evol. 2020;10: 3463–3476. doi:10.1002/ece3.6144

17. Benson DA, Cavanaugh M, Clark K, Karsch-Mizrachi I, Lipman DJ, Ostell J, et al. GenBank. Nucleic Acids Res. 2012;41: D36–D42.

18. Grey EK, Bernatchez L, Cassey P, Deiner K, Deveney M, Howland KL, et al. Effects of sampling effort on biodiversity patterns estimated from environmental DNA metabarcoding surveys. Sci Rep. 2018;8: 1–10.

19. Günther B, Knebelsberger T, Neumann H, Laakmann S, Arbizu PM. Metabarcoding of marine environmental DNA based on mitochondrial and nuclear genes. Sci Rep. 2018;8: 1–13.

20. Stoeckle MY, Das Mishu M, Charlop-Powers Z. Improved environmental DNA reference library detects overlooked marine fishes in New Jersey, United States. Front Mar Sci. 2020;7: 226.

**Supplemental Figures**


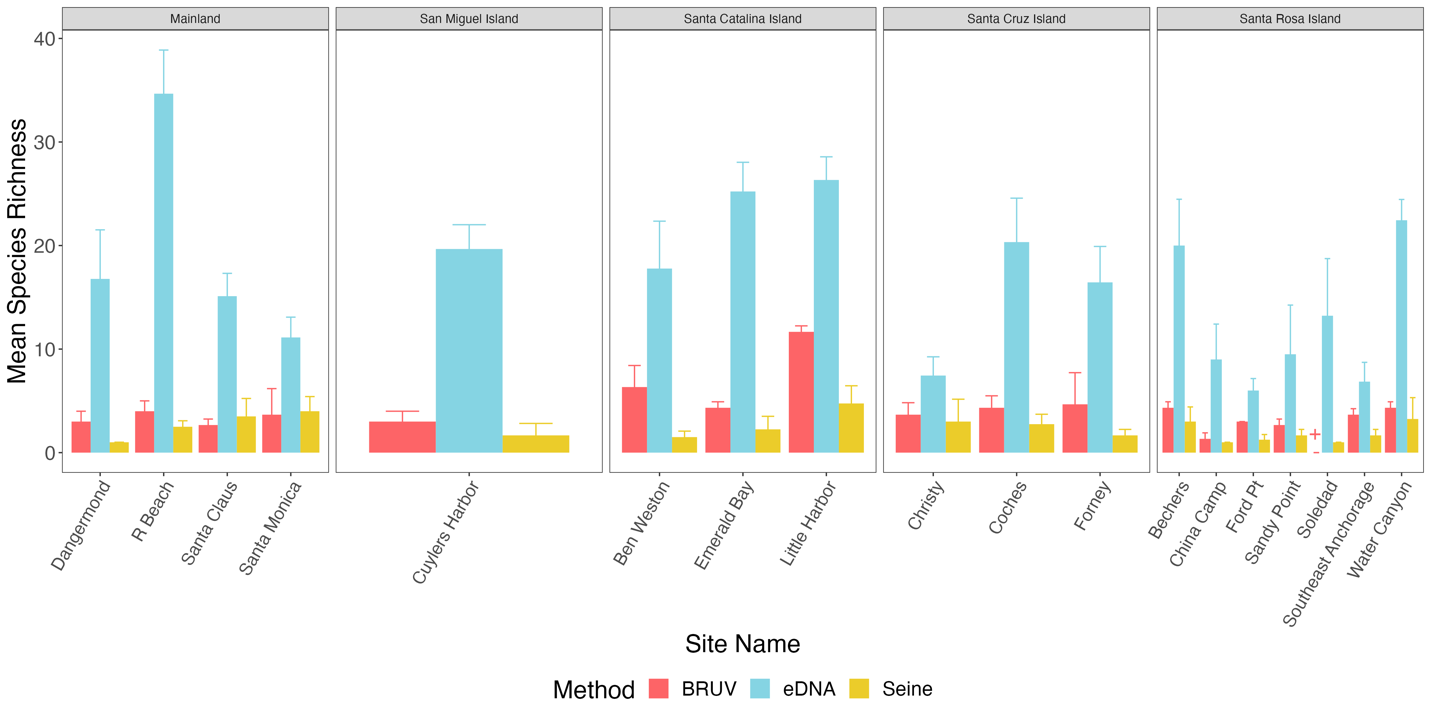


**Figure S1. eDNA Detects More Species than BRUV and Seine Surveys**

eDNA detected a significantly greater number of species (35.89  ± 10.31 SD species per site [range 13-58 species]) than the combined BRUV and seine survey results at all sites (ANOVA, p < 0.001). At the majority of sites, BRUV survey detected a significantly greater number of species (15.88 ± 8.91 SD species per site [range 5-35 species]) than seine (5.71 ± 2.58 SD species per site [range 0-10 species]) (ANOVA, p < 0.005). At Soledad, we failed to deploy BRUV, therefore we have no BRUV species data at that site(+).


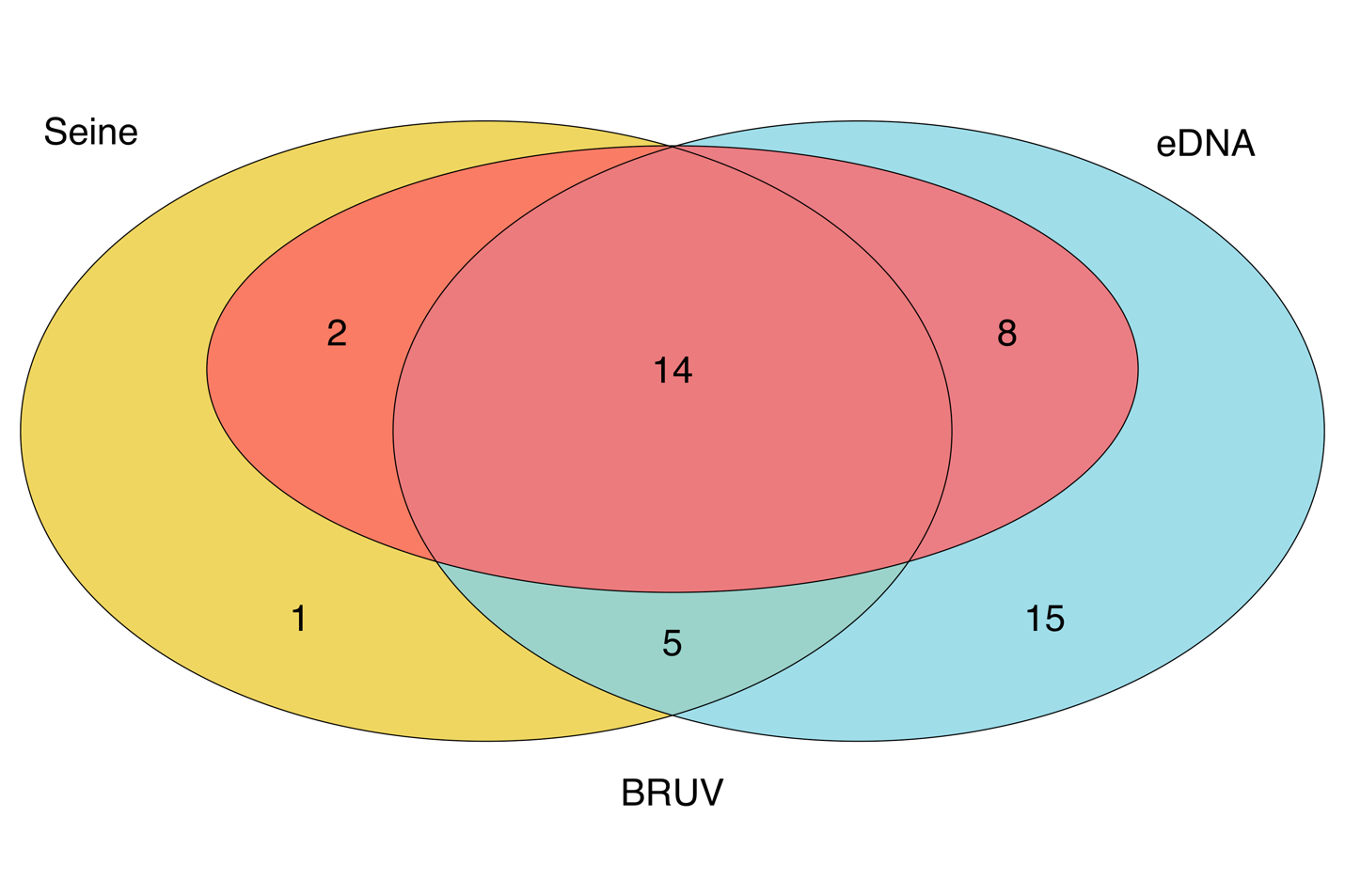


**Figure S2: Venn Diagram of eDNA, Seine, and BRUV Species Detections of Surf Zone Species**

Surf zone habitat association was determined using habitat descriptions from FishBase.org and previous literature (Allen and Pondella II 2006, Froese and Pauly 2010, Kells et al. 2016)


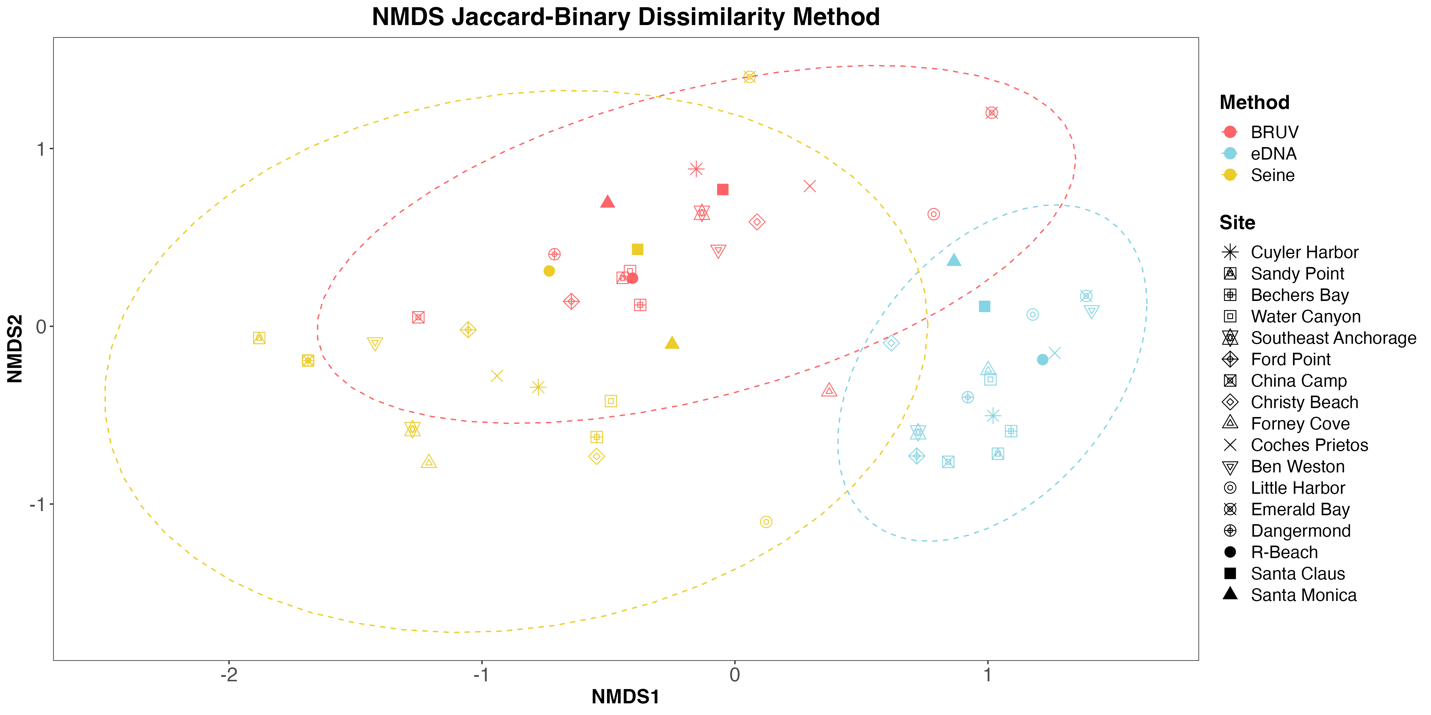


**Figure S3: NMDS ordination of fish assemblages detected by each method**

Jaccard-Binary dissimilarities of fish assemblages were calculated between all methods. Fish community structure clusters by method (color) (NMDS, Stress = 0.147). Method explained 41.5% of the variation and Site explained 28.8% of the variation in fish community assemblages (PERMANOVA, p <0.0001). We found no significant difference in the homogeneity of dispersions between methods or sites (*betadisper* p >0.5)


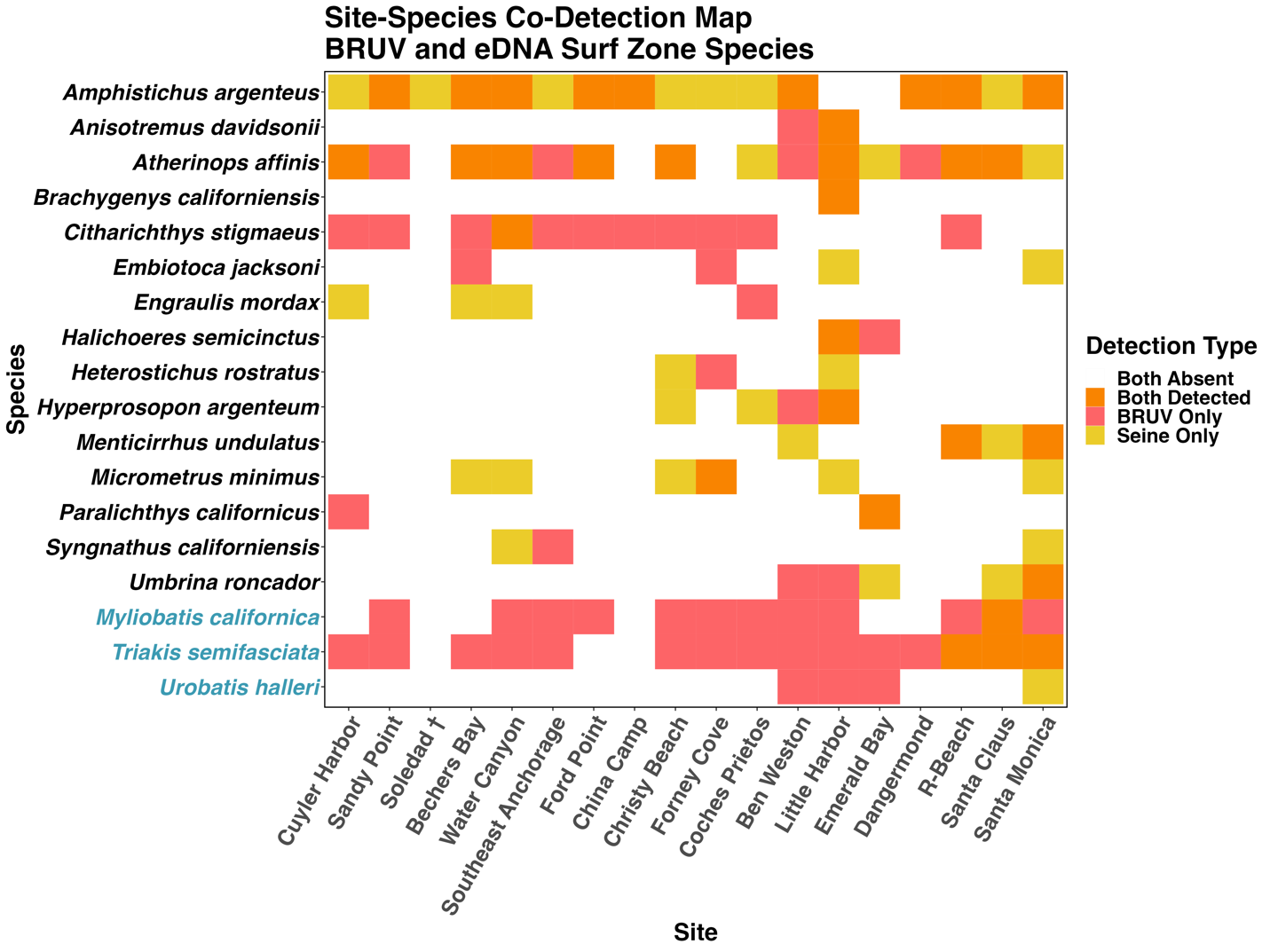


**Figure S4: Heatmap of surf zone fishes jointly detected between seine and BRUV surveys**

Teleost species in black font and elasmobranch species in blue font.

**
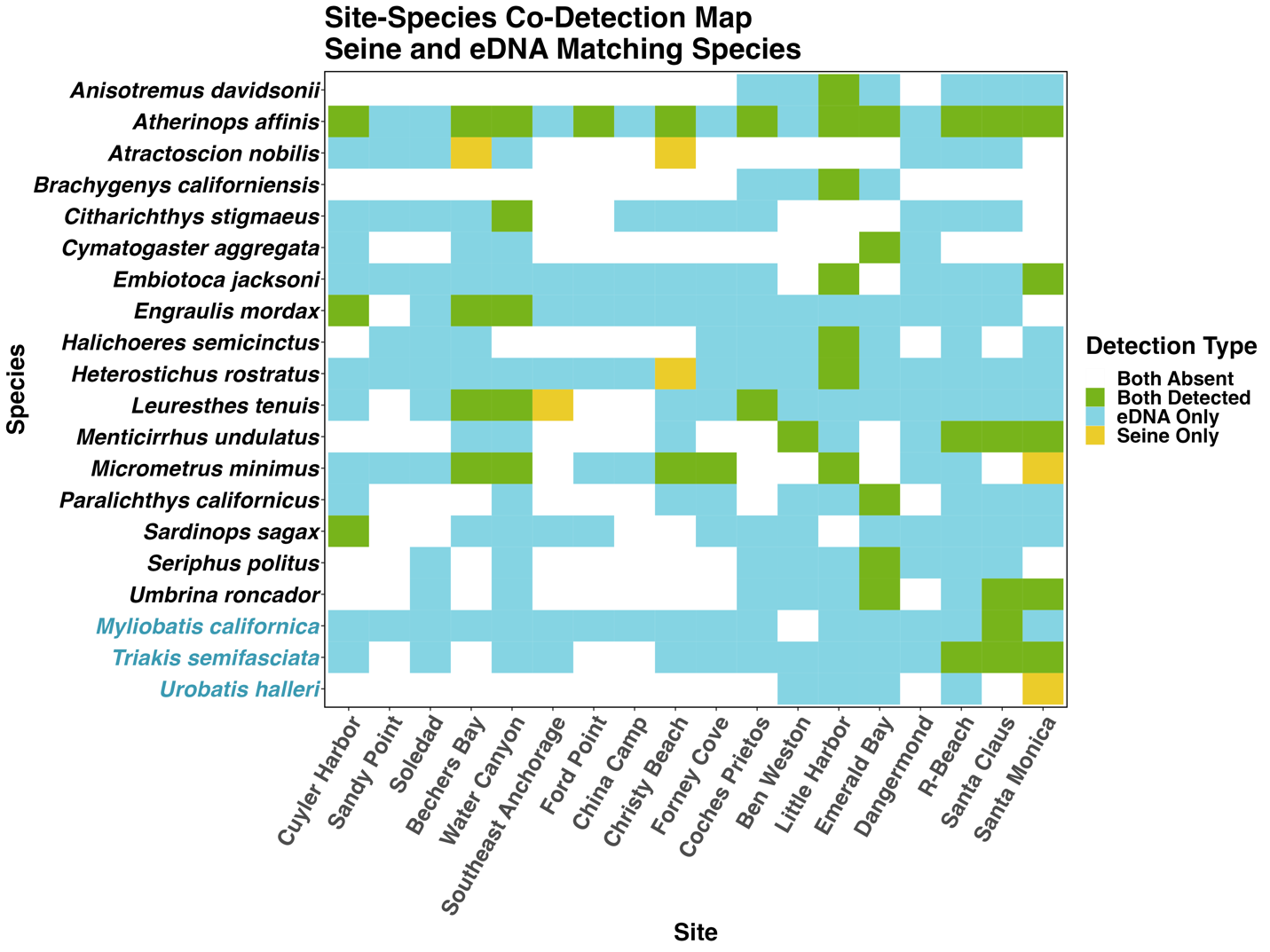
**

**Figure S5: Heatmap of surf zone fishes jointly detected between eDNA and seine surveys**

Teleost species in black font and elasmobranch species in blue font.


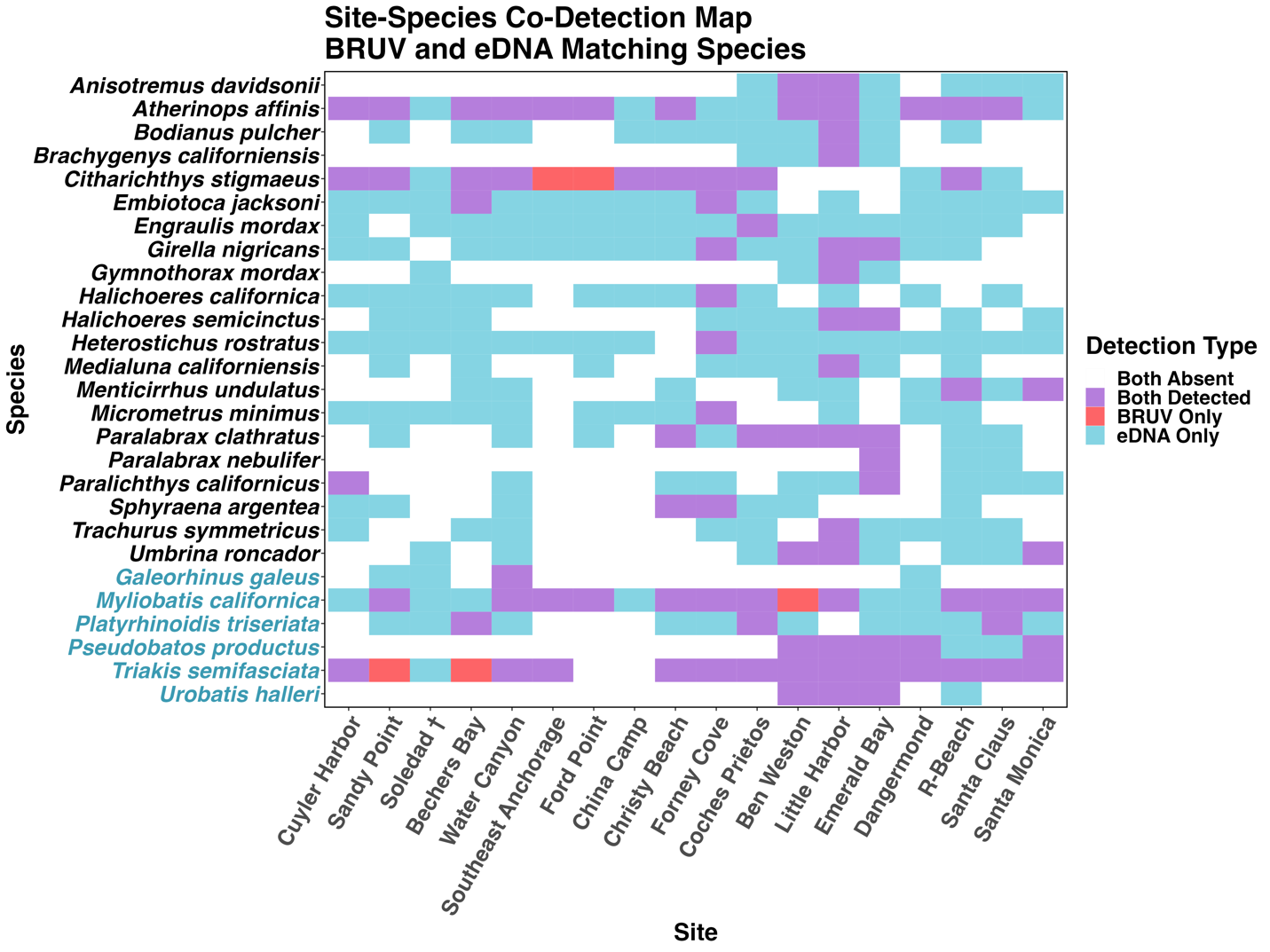


**Figure S6: Heatmap of surf zone fishes jointly detected between eDNA and BRUV surveys**

Teleost species in black font and elasmobranch species in blue font.


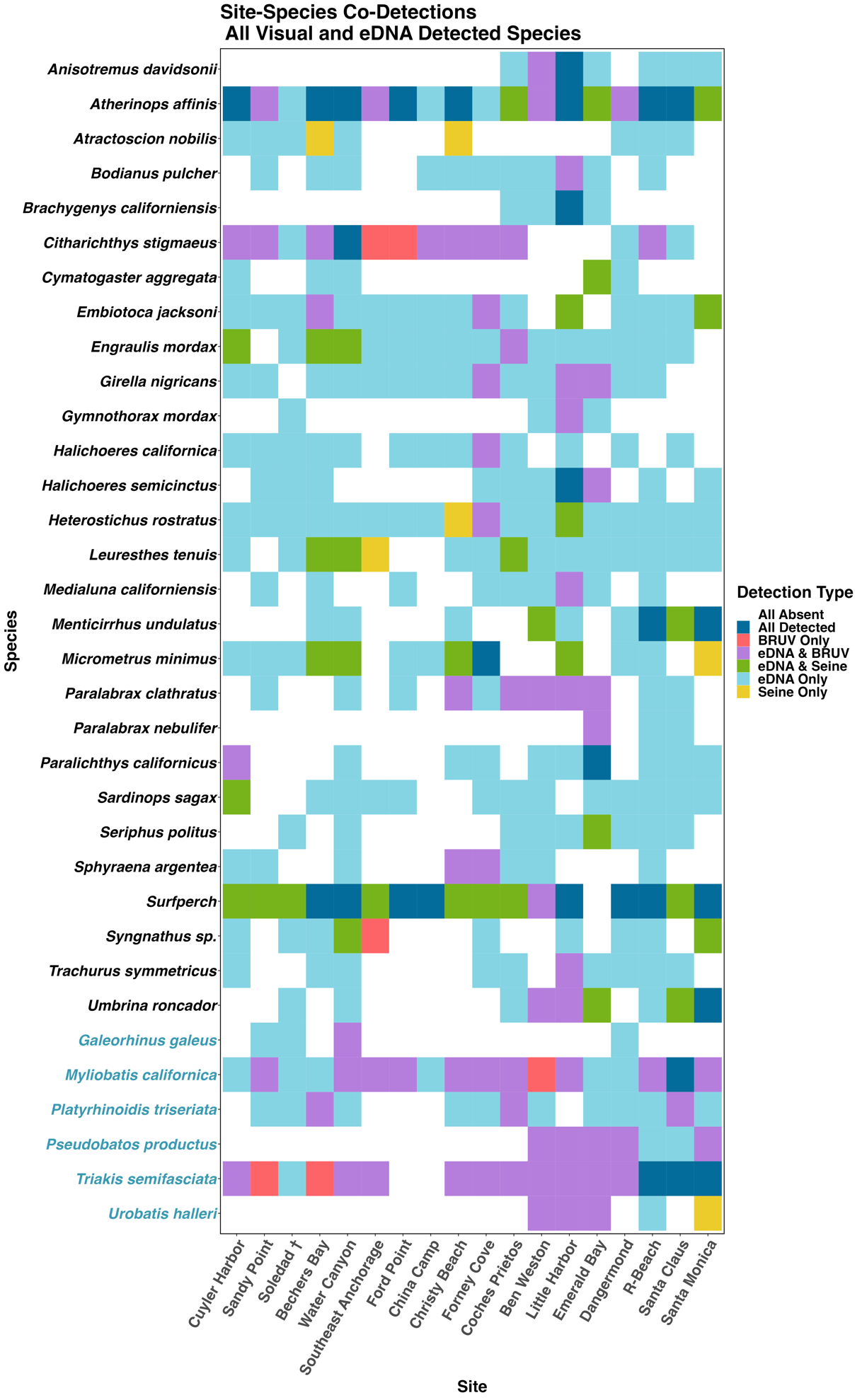


**Figure S7: Heatmap of fishes jointly detected by all surveys**

Teleost species in black font and elasmobranch species in blue font. Surfperchs and Syngnathus sp. with identical 12S barcodes were grouped across each method.


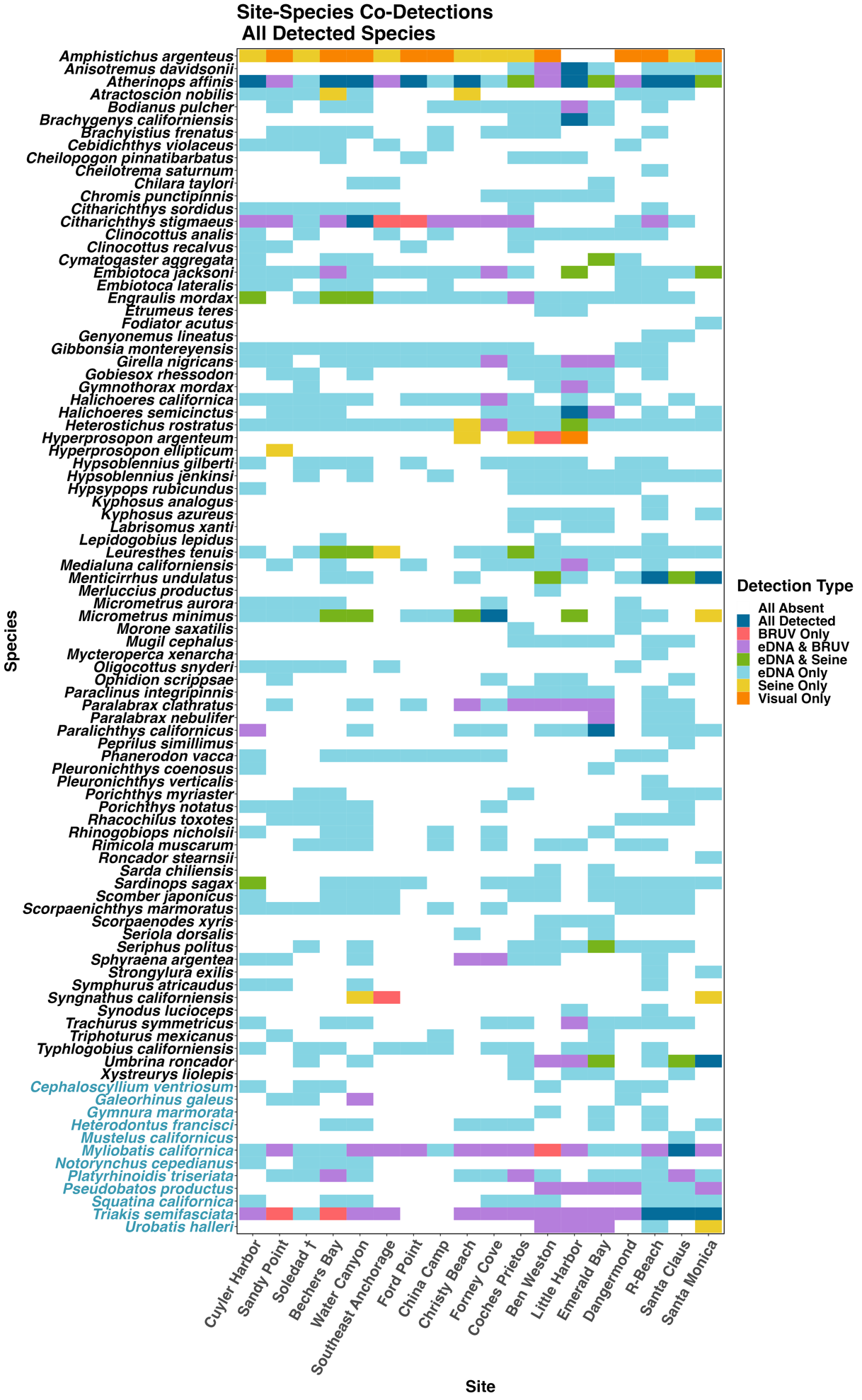


**Figure S8: Heatmap of fishes detected by all surveys**

Teleost species in black font and elasmobranch species in blue font.

**
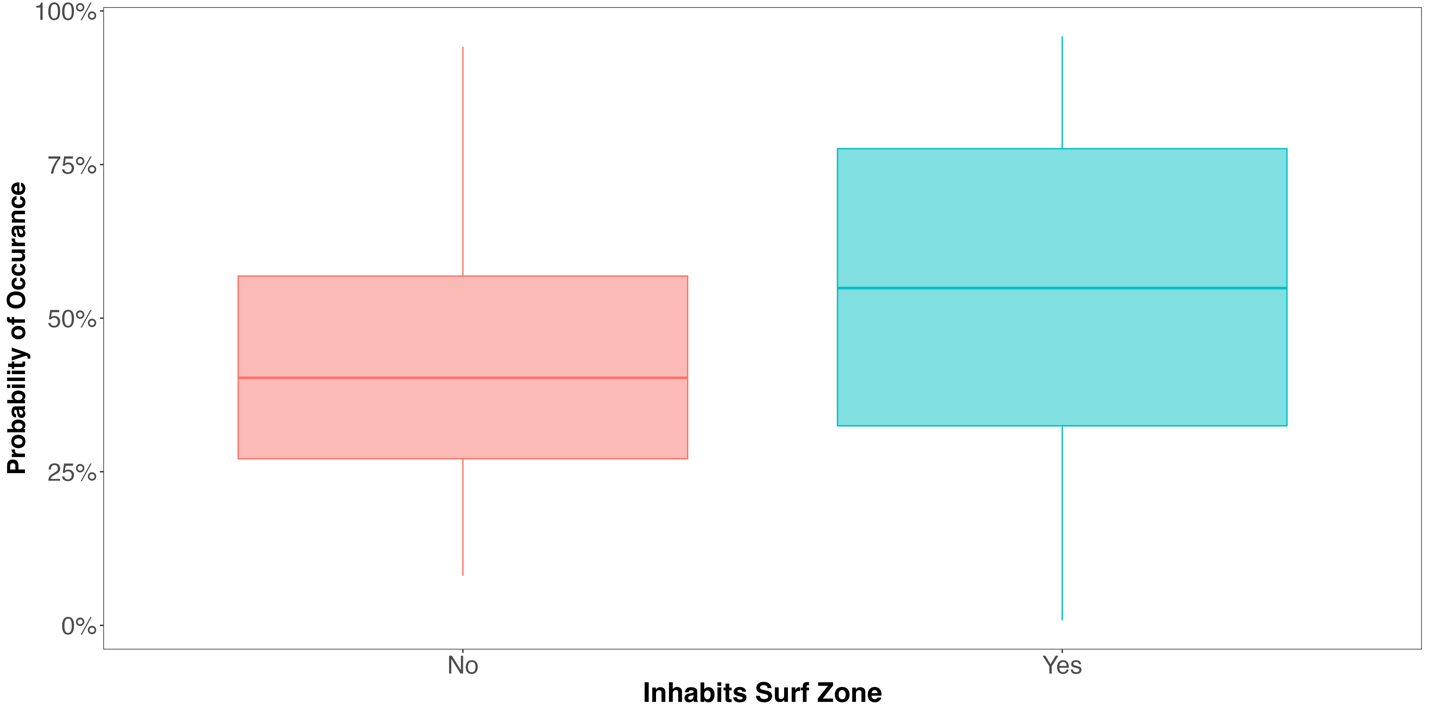
**

**Figure S9: Probability of Occupancy of Surfzone Species from eDNA Methods**


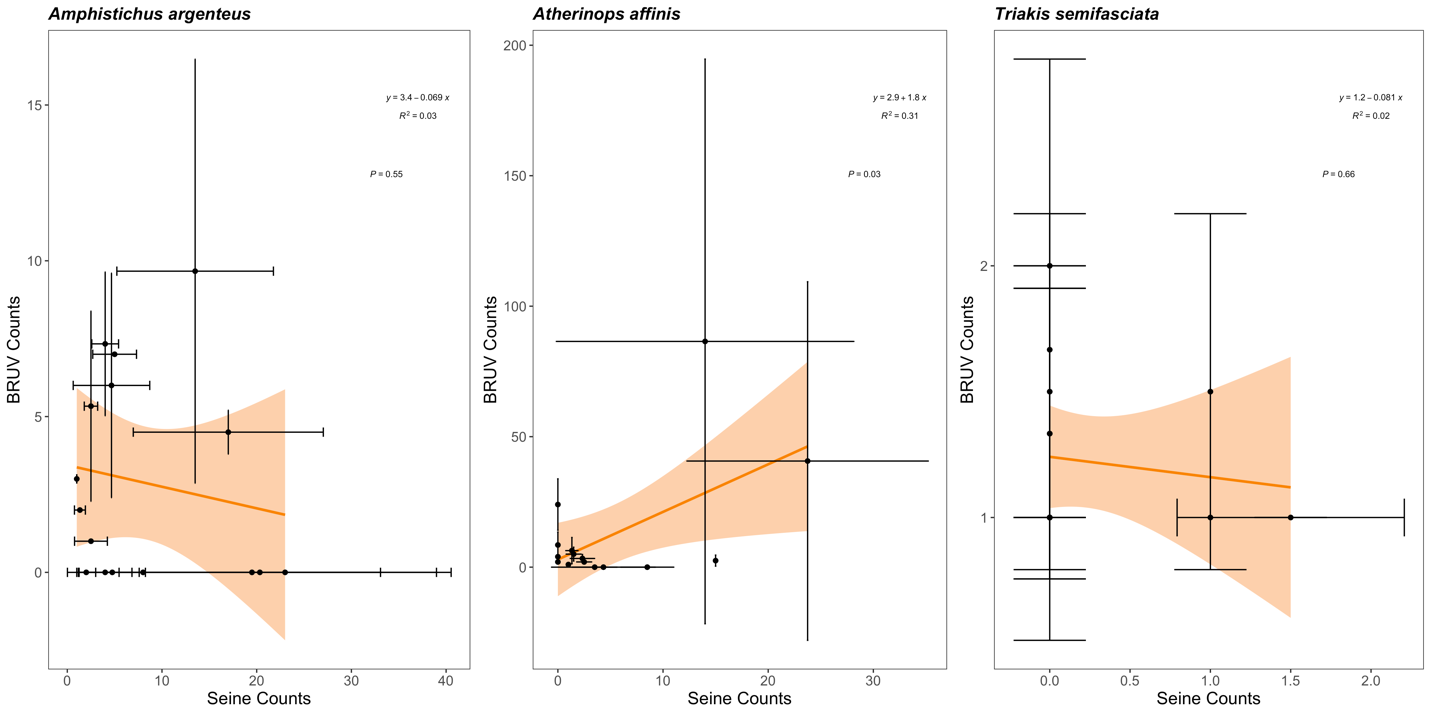


**Figure S10: Relative Abundance Comparisons of BRUV Counts vs. Seine Counts**

Relative abundance comparisons were only conducted for species with at least 3 joint site detections by both methods. Points are mean values and error bars represent standard deviations. Best fit of linear regressions are colored lines and the shaded region represents the 95% confidence interval.


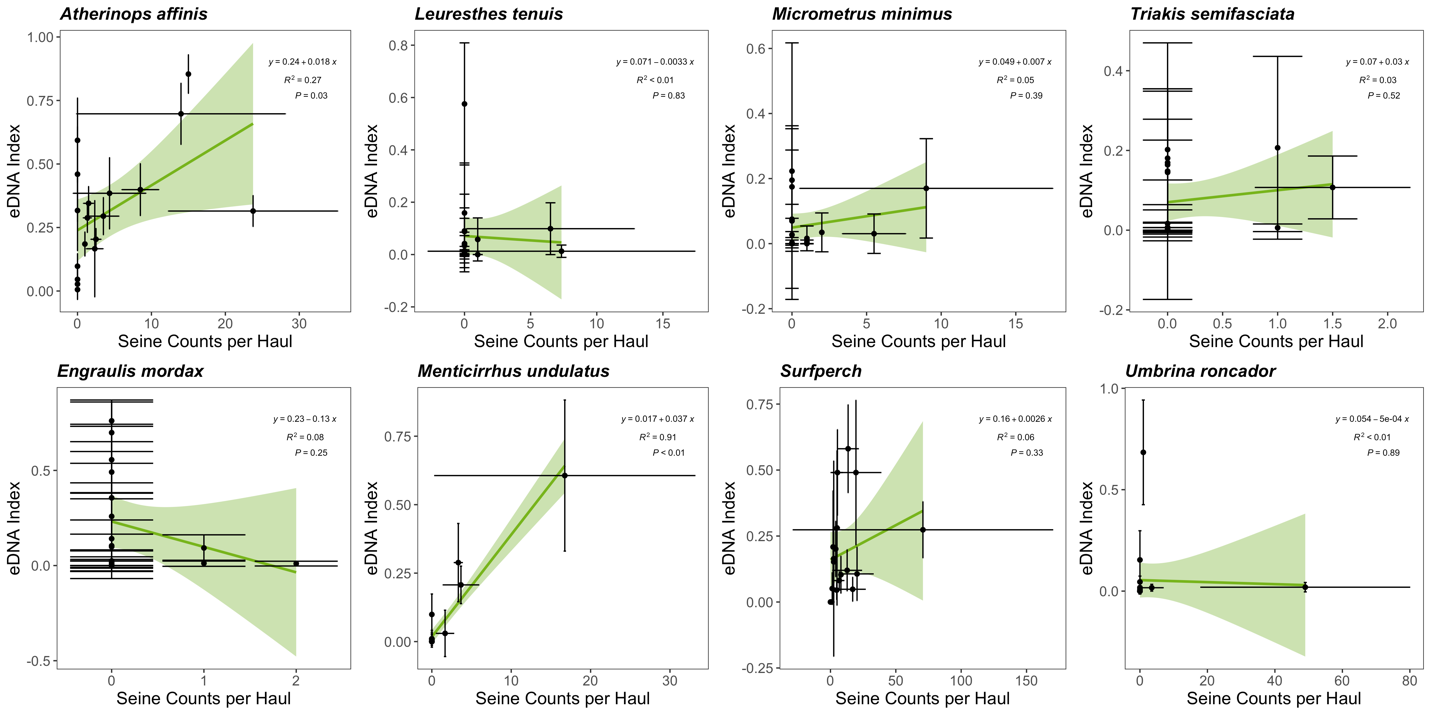


**Figure S11: Relative Abundance Comparisons of eDNA Index vs. Seine Counts**

Relative abundance comparisons were only conducted for species with at least 3 joint site detections by both methods. Points are mean values and error bars represent standard deviations. Best fit of linear regressions are colored lines and the shaded region represents the 95% confidence interval.


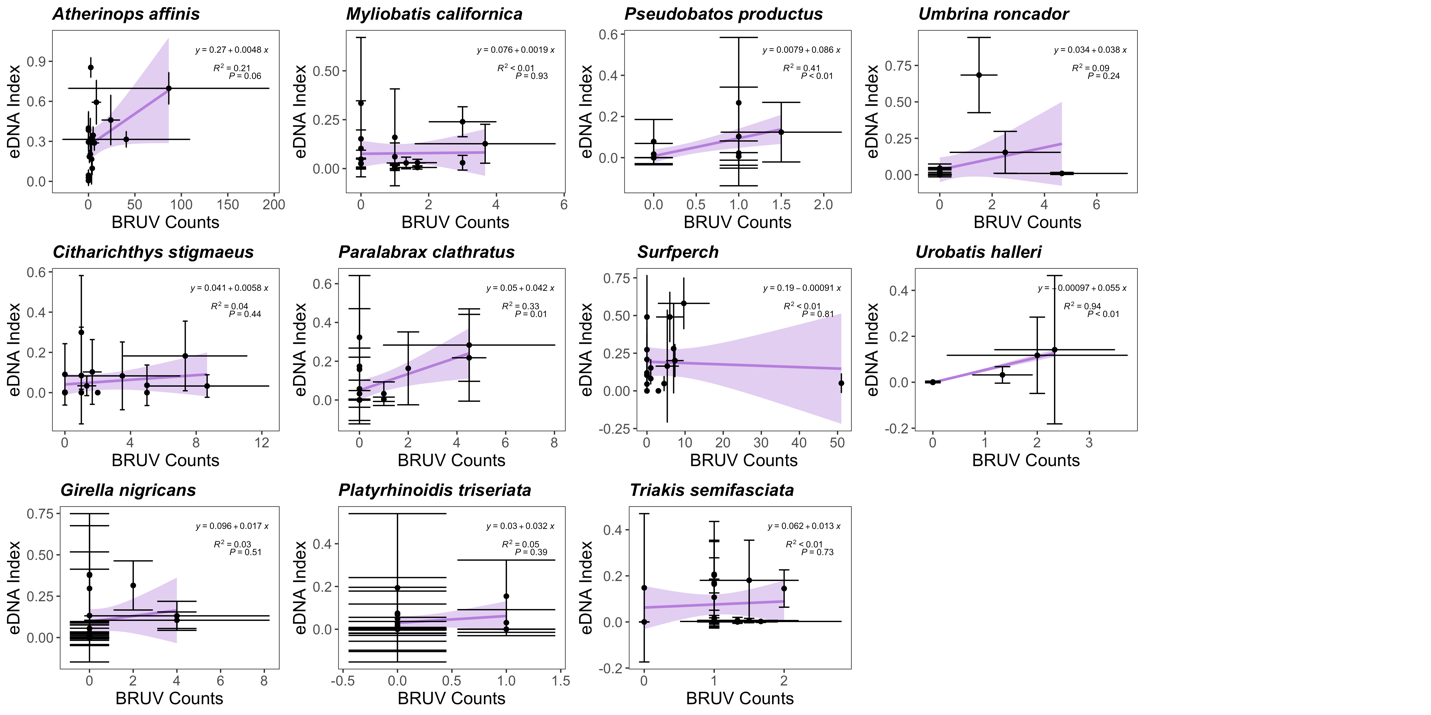


**Figure S12: Relative Abundance Comparisons of eDNA Index vs. BRUV Counts**

Relative abundance comparisons were only conducted for species with at least 3 joint site detections by both methods. Points are mean values and error bars represent standard deviations. Best fit of linear regressions are colored lines and the shaded region represents the 95% confidence interval.
